# The Development of Three-DNA Methylation Signature as a Novel Prognostic Biomarker in Patients with Colorectal Cancer

**DOI:** 10.1155/2020/3497810

**Published:** 2020-11-25

**Authors:** Shu Gong, Weijian Ye, Tiankai Liu, Shaofen Jian, Wenhua Liu

**Affiliations:** ^1^Department of Biological Sciences, Life Sciences College, Zhaoqing University, Zhaoqing 526061, China; ^2^Chengdu 23 Mofang Biotechnology Co., Ltd., Chengdu 610000, China; ^3^School of Life Science, Guizhou Normal University, Guizhou 550025, China

## Abstract

**Aims:**

The prognosis of colorectal cancer (CRC) remains poor. This study aimed to develop and validate DNA methylation-based signature model to predict overall survival of CRC patients.

**Methods:**

The methylation array data of CRC patients were retrieved from The Cancer Genome Atlas (TCGA) database. These patients were divided into training and validation datasets. A risk score model was established based on Kaplan-Meier and multivariate Cox regression analysis of training cohort and tested in validation cohort.

**Results:**

Among total 14,626 DNA methylation candidate markers, we found that a three-DNA methylation signature (NR1H2, SCRIB, and UACA) was significantly associated with overall survival of CRC patients. Subgroup analysis indicated that this signature could predict overall survival of CRC patients regardless of age and gender.

**Conclusions:**

We established a prognostic model consisted of 3-DNA methylation sites, which could be used as potential biomarker to evaluate the prognosis of CRC patients.

## 1. Introduction

Colorectal cancer (CRC) is the third leading cause of cancer death worldwide 1. Despite recent development of early diagnosis and treatment techniques, the 5-year survival rate of CRC patients is unsatisfactory. Currently, prognostic models for CRC based on some characteristics such as age and gender are not precise. The ability to distinguish high-risk CRC patients may help clinical trials to demonstrate clinical benefits 2. Therefore, highly specific and sensitive predictive prognostic biomarkers are urgently needed for accurate prediction of patient survival, which may provide important guide to estimate treatment outcomes.

Recent evidence shows that epigenetic markers such as DNA methylation have the potential as a variety of biomarkers in disease diagnosis and prognosis predication 3–5. The abnormal DNA methylation may be present commonly in tumors and can be utilized as one of the earliest distinguishing molecular characteristics in CRC. Therefore, building the novel DNA methylation prognostic signatures to distinguish high- and low-risk CRC patients is urgently needed, which can be helpful for the stratification of treatment and personalized therapy.

Up to now, the use of genome-wide methylation analysis for CRC is limited by the large sets of DNA methylation data and complex statistical analysis. It is also difficult for the reproducibility with other independent factors. In this study, we analyzed colon adenocarcinoma (*COAD*) samples with 450k DNA methylation array to identify a prognostic panel for CRC, using the Cancer Genome Atlas (TCGA) dataset. Next, we built 3-DNA methylation biomarkers model associated with patient survival by using the methylation level of all methylation markers related to CRC patients from TCGA. Finally, we applied the Kaplan-Meier method and the ROC analysis to build and evaluate the model performance. Our results showed that the 3-DNA methylation biomarkers could provide the high accuracy performance to predict CRC patient survival and may be used as the novel prognostics markers.

## 2. Materials and Methods

### 2.1. Data Source from TCGA Dataset

This is a bioinformatics analysis study and no ethical statement was required. DNA methylation data and the related clinical information including tumor stage, survival status, and survival time for patients were downloaded from TCGA-COAD project (TCGA, https://cancergenome.nih.gov/). The TCGA-COAD methylation data were obtained using Illumina Human Methylation 450k BeadChip (Illumina Inc., CA, USA). Total 480 COAD tissue samples and 41 adjacent normal colon tissue samples were included in the TCGA-COAD cohort.

### 2.2. Data Analysis

Only samples with complete clinical data were selected to analyze the correlation of DNA methylation. Duplicated clinical information samples could be removed. Ultimately, 457 samples including cases and normal colon tissue were included in this study, and the related clinical information for each sample was obtained from the database. We split the data into training dataset (70% of the entire dataset) and validation dataset (30% of the entire dataset). We applied the training dataset for model building and identifying the prognostic biomarkers and applied the validation dataset for checking the accuracy of the model.

### 2.3. Statistical Analysis

All statistical analyses were conducted using the R statistical package. The univariate Cox regression survival analysis was conducted in the training dataset to identify methylation markers significantly (*p* < 0.05) correlated with patient survival as candidate markers, which were put for further multivariate cox regression analysis. Three markers were selected from the candidates to construct the final model. The AUC value was used to measure predictive performance of models; the higher the AUC value is, the more reliable the model is. The prognostic risk scores for each patient were calculated based on this formula, and the patients were separated into “low-risk” and “high-risk” groups using the median risk score as the cutoff point. Kaplan-Meier survival analysis was performed to calculate the cumulative survival time and compare the differences in the survival time between the two groups. The ROC analysis was conducted with the “pROC” R package with the methylation biomarkers.

## 3. Results

### 3.1. The Clinical Information of Samples

We collect 457 samples in this study after the filtration from the TCGA-COAD project. The median survival time of all samples was 2,821 days. The gender of about 54% samples was male. The tumor histologic grade of all samples was assigned according to the World Health Organization criteria into stages I, II, III, and IV.

### 3.2. Identification of Signature Predicting CRC Prognosis

According to the univariate Cox regression model, the methylation levels were used as input variables in the training dataset, and 14,626 DNA methylation candidate markers (*p* < 0.05) were identified to be significantly associated with overall survival (OS) of COAD patients. Next, multivariate Cox regression was applied to screen the candidate markers, and 3 methylation markers (cg14660573, cg09353563, and cg00110724) were found to be the optimum prognostic model for predicting OS of COAD patients. The risk scoring formula of these 3 methylation sites was as follows: risk score = 0.09 × *β* value of cg14660573 + −0.04 × *β* value of cg09353563 + 2.06 × *β* value of cg00110724.

### 3.3. The Association between 3-DNA Methylation Signature Predicting Model and COAD Cohort in the Training and Validation Datasets

**Figure 1:**
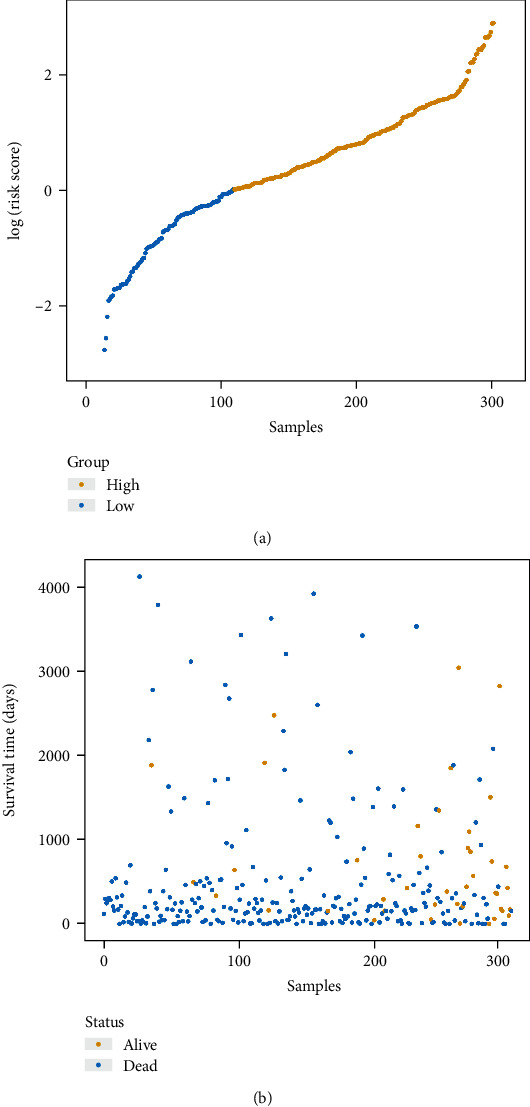
Risk score analysis of the 3-methylation signature and survival distribution by risk scores. (a) Risk score distribution of the 3-methylation signature. (b) Survival status distribution by risk scores.

**Figure 2:**
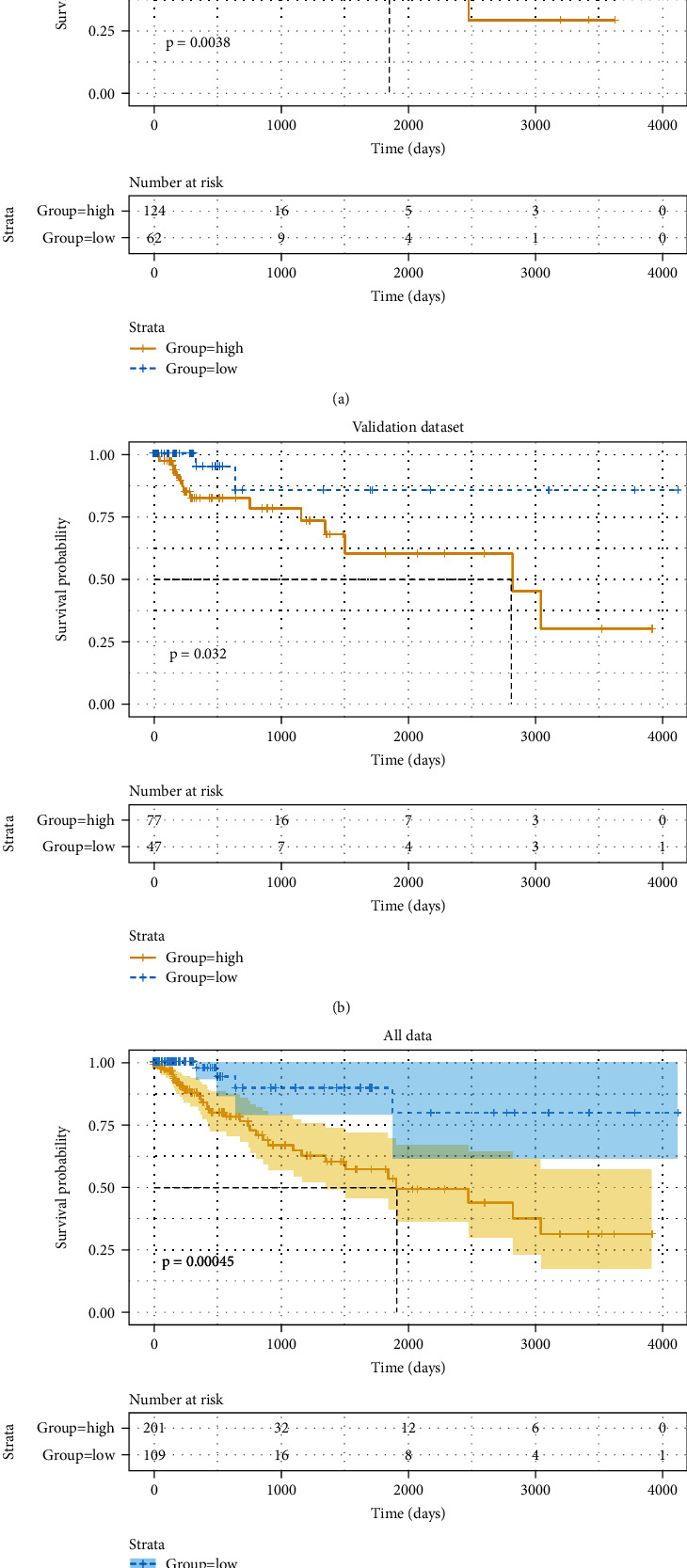
Kaplan-Meier curve of OS for high-risk and low-risk groups based on 3-methylation signature in training dataset (a), validation dataset (b), and all cohort dataset (c).

Kaplan-Meier analysis was performed to calculate the risk score of 3-DNA methylation markers; the distribution of risk score was shown in Figure 1(a). Then, the median of risk score was used as cut-off value to classify the dataset into high-risk group (*N* = 201) and low-risk group (*N* = 109). The mortality rate in the high-risk group was higher than that in the low-risk group (Figure 1(b)). The predicting model based on the training dataset demonstrated that samples in the high-risk group had a significantly lower survival rate, whereas the low-risk group had higher survival rate (*p* = 0.038, Figure 2(a)). Using this model, the same tendency was seen in the validation dataset and the all dataset (Figures 2(b) and 2(c)). These results indicated that 3-DNA methylation markers could distinguish the high-risk patients from the low-risk patients.

**Figure 3:**
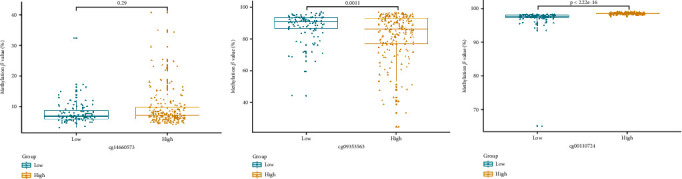
The differential methylation levels of cg14660573, cg09353563, and cg00110724 in high-risk and low-risk groups. Mann–Whitney *U* test was used to compare the differences between high-risk and low-risk groups.

In addition, differential levels of three methylation biomarkers were analyzed individually. The results showed that methylation levels of cg14660573 and cg00110724 were higher in the high-risk group than in the low-risk group, while methylation levels of cg09353563 were lower in the high-risk group than in the low-risk group (Figure 3). The three markers were related to the gene nuclear receptor subfamily 1 group H member 2 (NR1H2), Scribble Planar Cell Polarity Protein (SCRIB), and Uveal Autoantigen With Coiled-Coil Domains And Ankyrin Repeats (UACA), and the detailed information was listed in Table 1.

### 3.4. Validation of the 3-DNA Methylation Signature for Predicting CRC Prognosis

**Figure 4:**
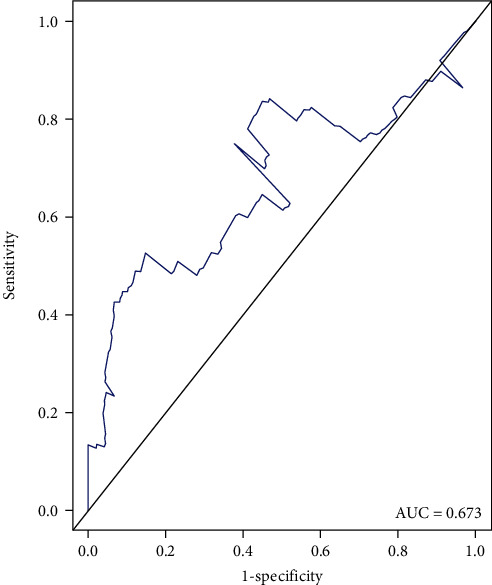
ROC curve showing the AUC of the 3-DNA methylation signature in predicting OS of CRC patients.

In order to examine the prediction performance of the 3-DNA methylation signature for CRC prognosis, ROC analysis was conducted to evaluate the sensitivity and specificity of the 3-DNA methylation signature in the validation dataset. The AUC of the 3-DNA methylation model was 0.673 (Figure 4), which indicated that this model could achieve high sensitivity (TPR: true positive rate) and specificity (FPR = 1-specificity: false-positive rate).

### 3.5. Prediction Performance of the 3-DNA Methylation Signature in CRC Patient Subgroups

**Figure 5:**
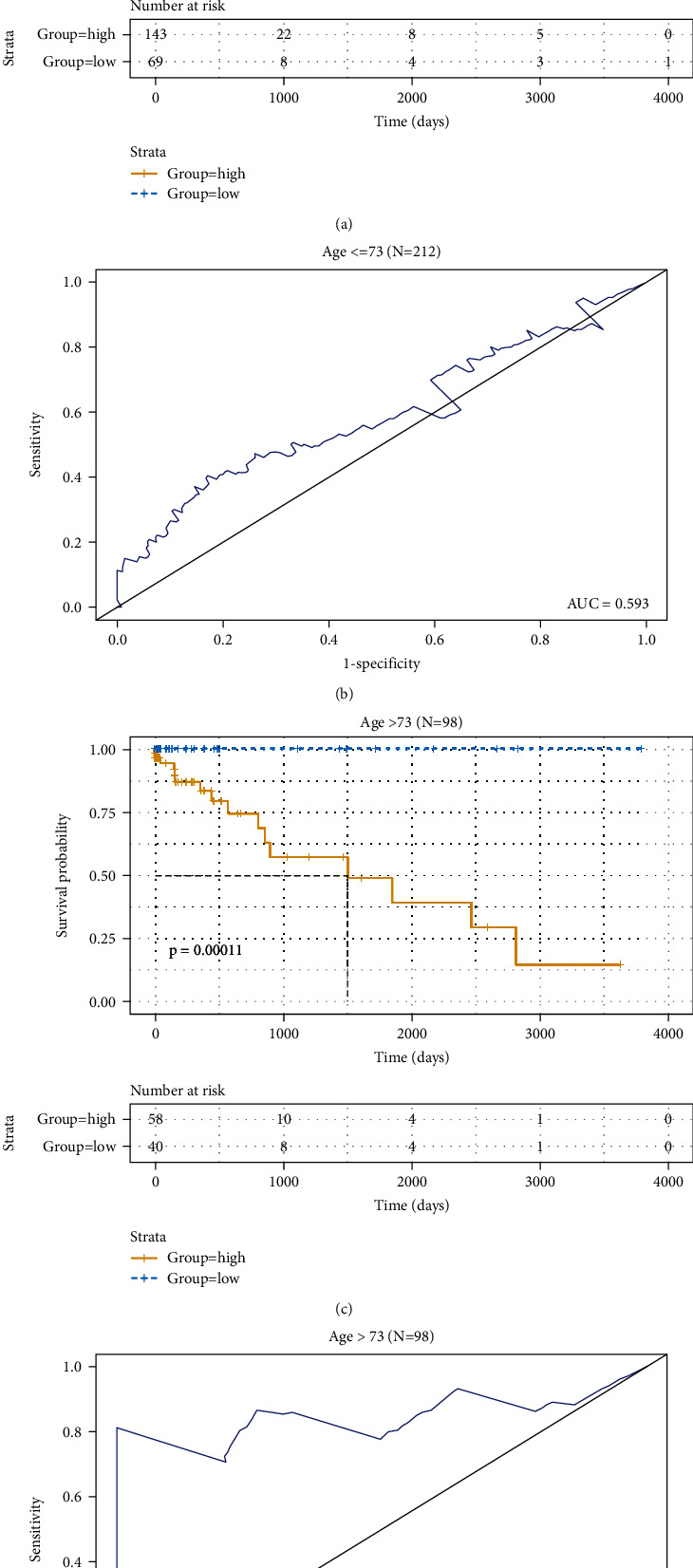
Kaplan-Meier and ROC analyses of patients in different age cohorts based on the age at initial diagnosis: ≤73 (*N* = 212, 68.4%), >73 (*N* = 98, 31.6%). (a, c) Kaplan-Meier analysis was performed to estimate the differences in OS between the low-risk and high-risk patients. (b, d) ROC curves of the 3-DNA methylation signature were used to demonstrate the sensitivity and specificity in predicting the OS of patients.

To examine whether the 3-DNA methylation signature could achieve high applicability in different clinical cohort, clinical features including the age and gender were used to regroup samples in the cohort, and then, each subgroup was further classified into high-risk and low-risk group using the 3-DNA methylation biomarkers model. First, patients were divided into two cohorts based on the ages at the initial diagnosis: <=73 (*N* = 212), >73 (*N* = 98). Kaplan-Meier curves showed that patients in the low-risk group had significantly longer OS in the younger age group (Figure 5(a)), and ROC analysis showed that the AUC value was 0.593 in this age group (Figure 5(b)). Similarly, Kaplan-Meier curves showed that patients in the low-risk group had significantly longer OS in the older age group (Figure 5(c)), and ROC analysis showed that the AUC value was 0.847 in this age group (Figure 5(d)).

**Figure 6:**
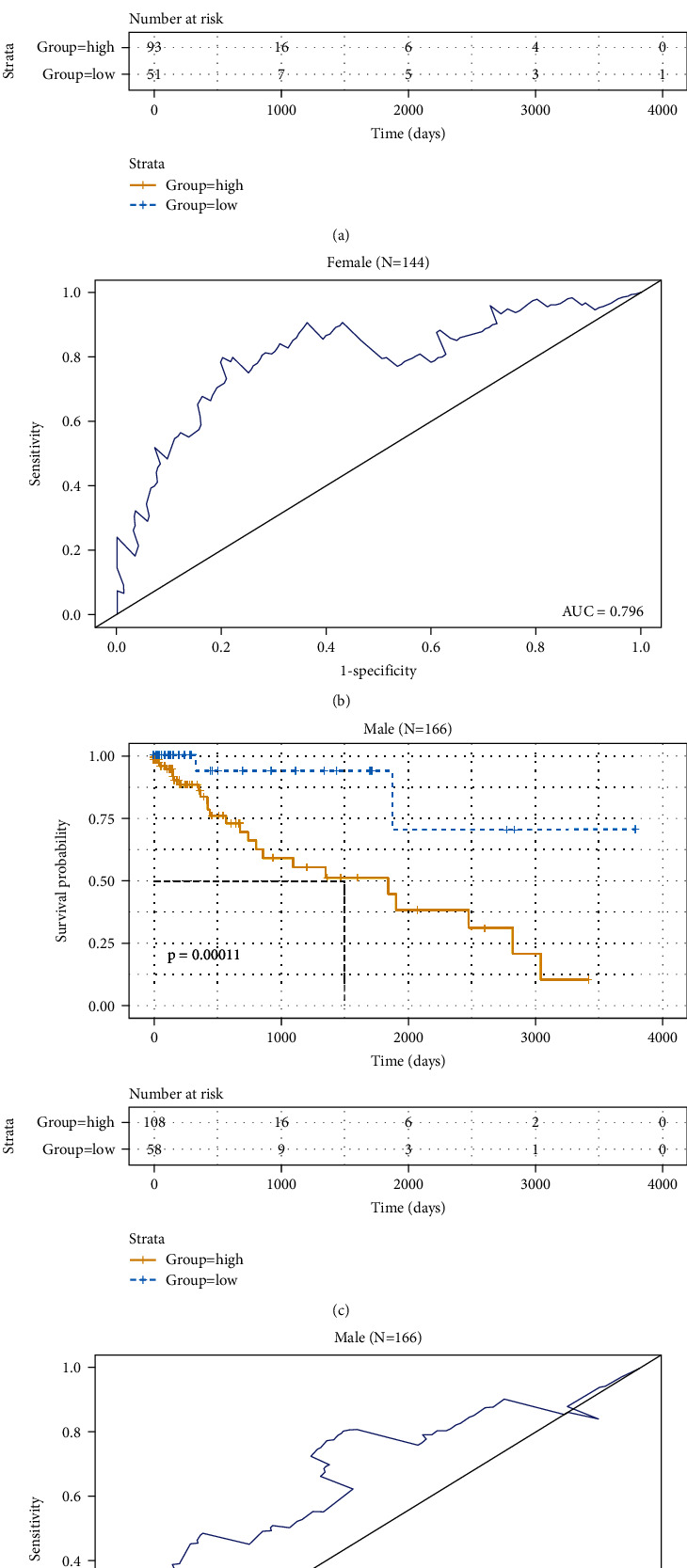
Kaplan-Meier and ROC analyses of patients in different gender cohorts, female (*N* = 144, 46.5%), and male (*N* = 166, 53.5%). (a, c) Kaplan-Meier analysis was performed to estimate the differences in OS between the low-risk and high-risk patients. (b, d) ROC curves of the 3-DNA methylation signature were used to demonstrate the sensitivity and specificity in predicting the OS of patients.

Next, patients were divided into two cohorts based on the gender. Kaplan-Meier curves showed that patients in the low-risk group had significantly longer OS in the female group (Figure 6(a)), and ROC analysis showed that the AUC value was 0.796 in the female group (Figure 6(b)). Similarly, Kaplan-Meier curves showed that patients in the low-risk group had significantly longer OS in the male group (Figure 6(c)), and ROC analysis showed that the AUC value was 0.678 in the male group (Figure 6(d)). Taken together, these data demonstrated that the 3-DNA methylation signature model could predict the survival status in COAD patients regardless of age and gender.

## 4. Discussion

The absence of highly effective and specific biomarkers for predicting the prognosis remains a challenge in clinical management of CRC patients. The recent rapid development of omics technologies such as genomes, transcriptomes, and proteomes provides new hope for establishing valuable prognostic models for CGC 6–10. However, the detection of large-scale expression levels of mRNAs, micro RNAs, long noncoding RNAs, or DNA polymorphisms could lead to variations due to different platforms and techniques used in the studies 11, 12. In contrast, epigenetic changes such as DNA methylation are reliable and specific cancer biomarkers that can be detected by PCR in blood and other body fluids samples easily obtained through noninvasive approach 13. In particular, evidence from several studies indicated that the combinations of several DNA methylation markers achieved higher sensitivity and specificity for cancer prognosis compared with individual DNA methylation marker 14–16. Therefore, in this study, we aimed to develop DNA methylation signature significantly associated with CRC prognosis with univariate and multivariate Cox proportional hazards regression analysis and the ROC analysis based on the genome-wide DNA methylation analysis. Our results demonstrated that the three-DNA methylation signature can perform well to distinguish high- and low-risk groups and that the risk score calculated by the three-DNA methylation signature could be a prognostic indicator for CRC patients.

Interestingly, the three-DNA methylation signature was related to the methylation in three genes encoding NR1H2, SCRIB, and UACA, respectively. NR1H2 is a member of the nuclear receptor superfamily and is composed of a central DNA-binding domain and C-terminal ligand-biding domain. NR1H2 could regulate glucose and cholesterol metabolism and is potentially involved in tumorigenesis 17. Interestingly, a recent study reported that NR1H2 mRNA levels were lower in CRC tissues compared to control 18. This result is consistent with our result that methylation levels of cg14660573 (NR1H2 gene) were higher in the high-risk group than in the low-risk group, suggesting that methylation of NR1H2 gene may contribute to CRC.

SCRIB is a membrane protein that plays a role in the maintenance of apical-basal cell polarity of the epithelial tissue and is implicated in tumorigenesis 19. Downregulation of SCRIB could disrupt the epithelial polarity and was strongly correlated with poor survival in prostate cancer patients 20. Consistently, with the potential tumor suppressor role of SCRIB, we found that methylation levels of cg00110724 (SCRIB gene) were higher in the high-risk group than in the low-risk group, suggesting that methylation of SCRIB gene would lead to the loss of tumor suppression and promote CRC.

UACA was first identified as an autoantigen in panuveitis patients and later studies showed that UACA expression was higher in lung adenocarcinoma and squamous cell carcinoma, independent of tumor grade 21. A recent study reported that the urine level of UACA was higher in prostate cancer patients and UACA could be a biomarker for prostate cancer 22. In this study, we found that methylation levels of cg09353563 (UACA gene) were lower in the high-risk group than in the low-risk group, indicating that the upregulation of UACA may promote CRC.

To our knowledge, this is the first study on 3-DNA methylation signature as prognostic biomarkers to predict CRC patient survival. While our model based on 3-DNA methylation signature showed high sensitivity and specificity to distinguish high-risk and low-risk patients, it is still early to conclude that our model is superior to traditional methods such as imaging to predict CRC patient outcomes. In addition, while our model incorporated age and gender to predict the OS of CRC patients, we did not analyze other clinical characteristics due to limited information in our study cohorts. In future studies, we need to detect the expression levels and predictive efficacy of three methylation sites in CRC cells and animal models of CRC to explore molecular mechanism underlying CRC progression.

In conclusion, in this study, we established a prognostic model consisted of 3 DNA methylation sites and validated the high sensitivity and specificity of this model in training and validation cohorts. Further studies are needed to confirm that the 3 DNA methylation signature could be used as a potential biomarker to evaluate the prognosis of CRC patients.

## Figures and Tables

**Table 1:** Three DNA methylation markers related to CRC risk.

ID	Chromosome location	Gene symbol	CGI coordinate	Feature type	*p* value (univariate)	*p* value (multivariate)
cg14660573	chr19:50376162-50376163	NR1H2	chr19:50376349-50377026	N_Shore	5e-06	0.004
cg00110724	chr8:143803681-143803682	SCRIB	chr8:143803099-143803933	Island	0.000208	0.013
cg09353563	chr15:70702096-70702097	UACA	chr15:70762559-70763891	None	1.2e-05	3e-04

## Data Availability

All data are available on request.
